# BET protein inhibition evidently enhances sensitivity to PI3K/mTOR dual inhibition in intrahepatic cholangiocarcinoma

**DOI:** 10.1038/s41419-021-04305-3

**Published:** 2021-10-29

**Authors:** Xiaolong Miao, Chen Liu, Yuancong Jiang, Yao Wang, Deqiang Kong, Zelai Wu, Xinyi Wang, Rui Tian, Xing Yu, Xuhang Zhu, Weihua Gong

**Affiliations:** 1 Department of Surgery, Second Affiliated Hospital of School of Medicine, Zhejiang University, Hangzhou, China; 2 College of Life Sciences and Medicine, Zhejiang Sci-Tech University, Hangzhou, China; 3 Department of Biochemistry, Zhejiang University, Hangzhou, China; 4 Department of head and neck Surgery, Zhejiang Cancer Hospital, Hangzhou, China; 5 Cancer Center, Zhejiang University, Hangzhou, China; 6 Liangzhu Laboratory, Zhejiang University Medical Center, 1369 West Wenyi Road, Hangzhou, China

**Keywords:** Cancer models, Targeted therapies

## Abstract

Intrahepatic cholangiocarcinoma (ICC), the second most common primary liver cancer, is a fatal malignancy with a poor prognosis and only very limited therapeutic options. Although molecular targeted therapy is emerged as a promising treatment strategy, resistance to molecular-targeted therapy occurs inevitably, which represents a major clinical challenge. In this study, we confirmed that mammalian target of rapamycin (mTOR) signaling is the most significantly affected pathways in ICC. As a novel phosphoinositide 3-kinase (PI3K)/mTOR dual inhibitor, BEZ235, exerts antitumour activity by effectively and specifically blocking the dysfunctional activation of the PI3K/serine/threonine kinase (AKT)/mTOR pathway. We generate the orthotopic ICC mouse model through hydrodynamic transfection of AKT and yes-associated protein (YAP) plasmids into the mouse liver. Our study confirmed that BEZ235 can suppress the proliferation, invasion and colony conformation abilities of ICC cells in vitro but cannot effectively inhibit ICC progression in vivo. Inhibition of PI3K/mTOR allowed upregulation of c-Myc and YAP through suppressed the phosphorylation of LATS1. It would be a novel mechanism that mediated resistance to PI3K/mTOR dual inhibitor. However, Bromo- and extraterminal domain (BET) inhibition by JQ1 downregulates c-Myc and YAP transcription, which could enhance the efficacy of PI3K/mTOR inhibitors. The efficacy results of combination therapy exhibited effective treatment on ICC in vitro and in vivo. Our data further confirmed that the combination of PI3K/mTOR dual inhibitor and BET inhibition induces M1 polarization and suppresses M2 polarization in macrophages by regulating the expression of HIF-1α. Our study provides a novel and efficient therapeutic strategy in treating primary ICC.

## Graphical abstract

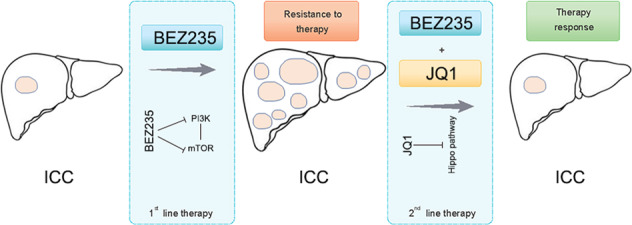

## Introduction

Cholangiocarcinoma (CCA) is a heterogeneous group of cancers with pathologic features of intra- or extrahepatic biliary tract differentiation [1]. Based on its anatomical origin, CCA is classified as intrahepatic, perihilar, or distal CCA [2]. Intrahepatic cholangiocarcinoma (ICC) is the second most common primary liver cancer, following hepatocellular carcinoma (HCC), and the incidence of ICC is increasing annually [1]. Established risk factors for ICC are hepatolithiasis, hepatitis virus, and hepatobiliary flukes [1]. The mortality rates of ICC are very high because patients with ICC are usually diagnosed at advanced stages, which excludes the majority of them from surgical treatment [3]. The efficacy of a gemcitabine/oxaliplatin-based regimen, a standard chemotherapy for advanced ICC, is rather limited [4]. However, the five-year overall survival (OS) rate of patients with intrahepatic cholangiocarcinoma (ICC) is still less than 5% [4, 5]. Therefore, the development of novel therapeutic strategies is in great demand.

The PI3K/AKT and mTOR signaling pathways play a key role in cell proliferation, survival, and metabolism and are two of the most commonly dysregulated oncogenic pathways in cancers, including renal cell carcinoma, breast cancer, mantle cell lymphoma, adult soft tissue cancer, and bone sarcomas [6]. BEZ235, an imidazo(4,5-c) quinoline derivative, exerts antitumour activity by effectively and specifically blocking the dysfunctional activation of the PI3K/AKT/mTOR pathway, inducing G [1] arrest [7].

BEZ235 suppresses the proliferation, migration and colony formation abilities of cancer cells and induces autophagy in tumorigenesis and tumor development [8]. However, single-agent BEZ235 typically leads to cytostasis [9], under which tumors can relapse due to the emergence of resistant cells that escape proliferative suppression [10]. The upregulation of yes-associated protein (YAP) and c-MYC might be one of the resistance mechanisms that allows proliferation under chronic PI3K/mTOR inhibition [10]. As a result, there is growing consensus that improved co-targeting strategies are warranted [11, 12].

A novel cell-permeable small molecule, JQ1, was reported to reversibly bind to bromodomains; disrupt the association of BET proteins, which are a family of epigenetic regulators with acetylated lysine in histones and transcription factors; repress oncogene expression; and eventually lead to the cessation of cancer cell growth [13]. JQ1 has been used as a new epigenetic therapeutic strategy for multiple cancers, especially advanced aggressive cancer types such as castration-resistant prostate cancer (CRPC), triple-negative breast cancer (TNBC), and nuclear protein in testis (NUT) midline carcinoma [6, 13, 14]. JQ1 is an inhibitor of c-Myc, which is a proto-oncogene overexpressed in most cancer cells [15–17]. In our previous study, the YAP/transcriptional coactivator with PDZ-binding motif (TAZ) and Notch signaling pathways were reported to be suppressed by JQ1 [18].

There has been no study investigating the effect of combination therapy in ICC cell lines and primary malignancy animal models. We found that JQ1 can suppress the expression of YAP and c-MYC, which are involved in resistance to PI3K/mTOR inhibition. Our study therefore provides a novel therapeutic strategy in treating ICC.

## Results

### The PI3K/mTOR dual inhibitor inhibits the function of ICC cells in vitro

**Fig. 1:**
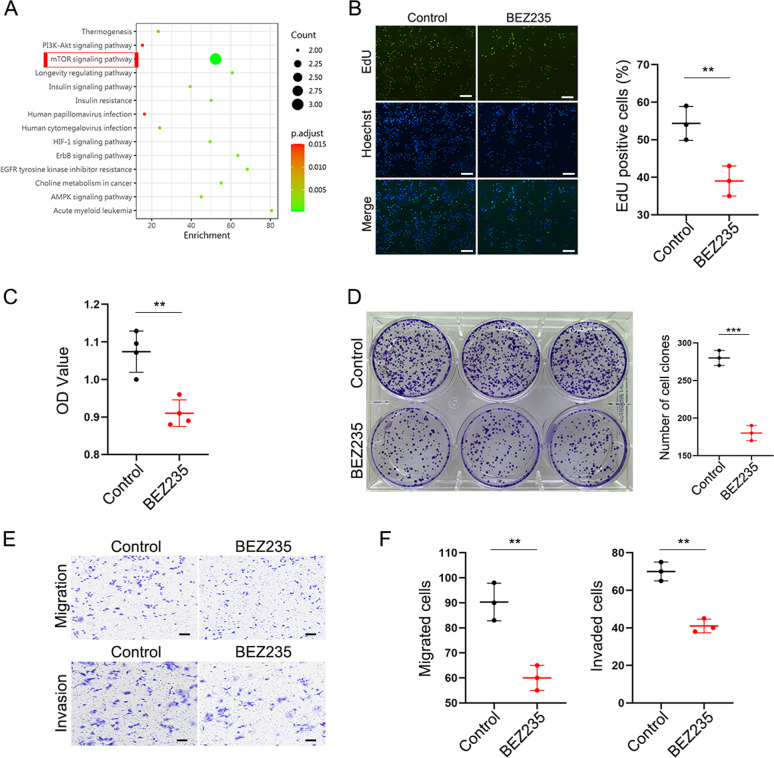
The PI3K/mTOR dual inhibitor inhibits the function of ICC cells in vitro. **A** KEGG pathway analysis showing the activated/phosphorylated protein between human intrahepatic biliary epithelial cell line (HIBEpiC) and human cholangiocarcinoma cell line (RBE). **B** EdU assay, **C** Cell Counting Kit-8 assay and **D** colony formation assay showing the cell proliferation ability in RBE cells treated with DMSO or BEZ235 (100 nM) for 24 h. Magnification, ×100; scale bar, 100 μm. **E**, **F** Transwell assay showing the migration potential and invasion potential of BEZ235 and DMSO treatment. Magnification, ×100; scale bar, 100 μm. The data are shown as the mean ± SEM (*p < 0.05, **p < 0.01, ***p < 0.001).

We compared the activated/phosphorylated proteins between a human intrahepatic biliary epithelial cell line (HIBEpiC) and a human cholangiocarcinoma cell line (RBE) by KEGG (Kyoto Encyclopedia of Genes and Genomes) pathway enrichment analysis. KEGG pathway analysis established mTOR signaling among the most significantly affected pathways (Fig. 1A). RBE cells were treated with DMSO or BEZ235 (100 nM) for 24 h. The EdU assay showed a higher percentage of EdU-positive (proliferating) cells in the control group than in the BEZ235 treatment group (*p* = 0.0079) (Fig. 1B). CCK-8 assays showed a similar result to the EdU assay: BEZ235 significantly inhibited RBE cell proliferation (*p* = 0.0024) (Fig. 1C). In addition, a colony formation assay was performed, which showed a significant decrease in the BEZ235 treatment group compared to the control group (*p* = 0.0003) (Fig. 1D). In order to prove the universality and reliability of this conclusion, the repetitions were carried out independently (Supplemental Fig. 1A). Transwell assays revealed the inhibitory effect of BEZ235 treatment on RBE cells, including a significant decrease in cell migration (*p* = 0.0043) and invasion (*p* = 0.0012) (Fig. 1E, F). The above results validated that BEZ235 suppressed the proliferation of RBE cells in vitro.

### Therapeutic efficacy of the PI3K/mTOR dual inhibitor in the treatment of ICC in vivo

**Fig. 2:**
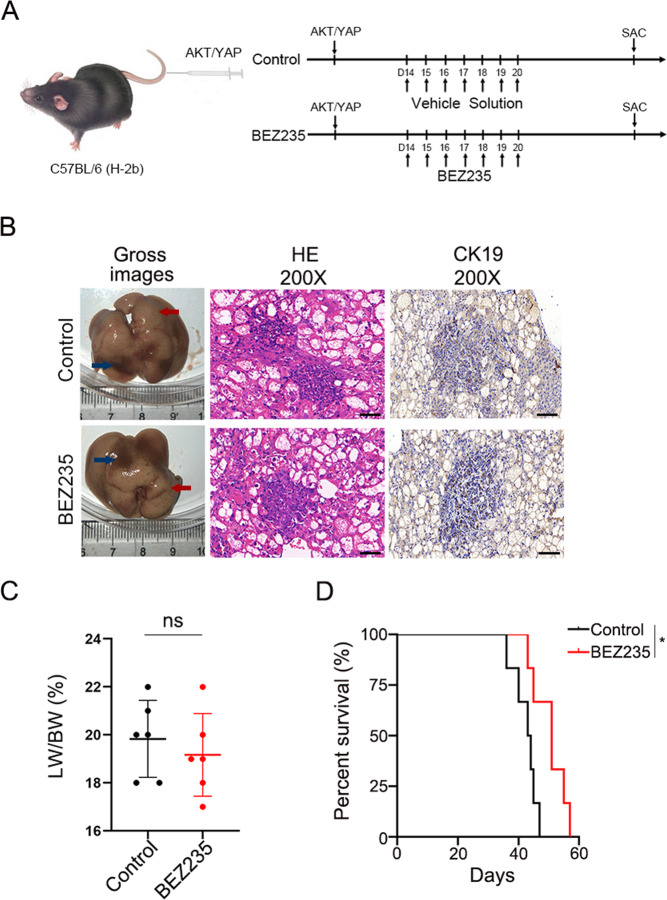
Therapeutic efficacy of the PI3K/mTOR dual inhibitor in the treatment of ICC in vivo. **A** Schematic of the experimental procedure for BEZ235 treatment. Mice were treated with either vehicle or BEZ235 (30 mg/kg) at 14–20 days (twice a day) after plasmids transfection by HTVi. All mice were sacrificed 6 weeks after oncogene transfection for phenotypic analysis. **B** Gross images, H&E staining and CK19 IHC staining of livers from vehicle- and BEZ235-treated AKT/YapS127A mice. Magnification, ×200; scale bar, 50 μm. **C** Tumor burdens were calculated by LW/BW ratio. The data are expressed as the means ± S.E.M (*n* = 6 per group, NS, *P* ≥ 0.05) for any other groups versus the control group. Abbreviation: SAC, sacrificed. **D** Survival curve of AKT/YapS127A mice treated with vehicle or BEZ235. Quantified data are presented as mean ± SE (*P < 0.05).

Due to the high expression of AKT and YAP in human ICC [19], we delivered plasmids (AKT/YAP S127A) for sleeping beauty transposase via hydrodynamic injection to generate the ICC model. Consistently, this model was confirmed by cytokeratin 19 (CK19) immunohistochemical staining and histological analysis (Fig. 2B). The experimental strategy for BEZ235 administration is shown in Fig. 1A. Six weeks after the hydrodynamic injection, mice were sacrificed. We found no significant difference in treatment effect between the BEZ235 groups and the control groups by macroscopic examination and H&E staining of liver sections (Fig. 2B). In addition, there was no statistically significant difference in the liver weight to body weight (LW/BW) ratio (*p* = 0.1401) (Fig. 2C). There was a modest difference between the two groups in the survival rate (*p* = 0.0437) (Fig. 2D). However, the difference was not significant. Taken together, these results indicate that BEZ235 cannot effectively inhibit ICC progression in vivo.

### The PI3K/mTOR dual inhibitor increased c-Myc and YAP expression in vivo and in vitro

**Fig. 3:**
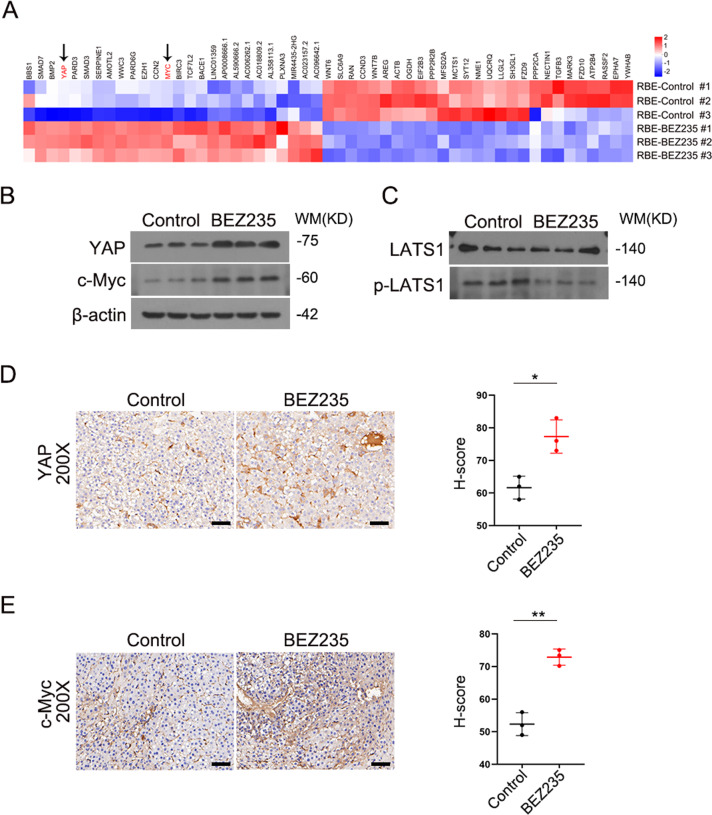
The PI3K/mTOR dual inhibitor increased c-Myc and YAP expression in vivo and in vitro. **A** the significant increase of MYC and YAP expression in BEZ235-treated RBE cells compare to DMSO-treated RBE cells by RNA-seq analysis. **B**, **C** The expression of YAP, c-Myc, LATS1 and p- LATS1 was detected in BEZ235-treated RBE cells and DMSO-treated RBE cells by western blotting for screening. **D**, **E** Representative immunostaining of YAP and c-Myc in tumor areas in liver sections. Magnification, ×200; scale bar, 50 μm. The data are shown as the mean ± S.E.M (*p < 0.05, **p < 0.01).

Accumulating evidence has suggested that the Hippo pathway plays an essential role in mediating resistance to cancer therapeutics [20]. Then, we detected a significant increase in MYC and YAP expression in BEZ235-treated RBE cells by RNA-seq analysis (Fig. 3A). Concurrently, increased levels of c-Myc and YAP protein expression were measured in BEZ235-treated RBE cells compared to dimethyl sulfoxide (DMSO)-treated RBE cells (Fig. 3B). Similarly, the same experimental western blotting results were also obtained with HuCCT1 cells and QBC939 cells (Supplemental Figs. 2A and 3A). The phosphorylation of LATS1 inhibits YAP/TAZ, which is the main effector of the Hippo pathway [21]. YAP transcribes c-Myc and promotes the expression of metabolic enzymes [22]. We confirmed that BEZ235 downregulated the phosphorylation of LATS1 in RBE cells. However, there was no significant difference in the protein level of total LATS1 (Fig. 3C). This is a novel mechanism by which BEZ235 upregulates c-Myc and YAP expression by suppressing the phosphorylation of LATS1. To confirm this inference, we treated RBE cells with Nitidine chloride. Previous evidence indicated that Nitidine chloride treatment significantly enhanced the level of p-LATS1 in cancer cells. The levels of p-LATS1 was markedly increased in response to Nitidine chloride treatment (Supplemental Fig. 4A). As depicted in Supplemental Fig. 3B, western blot analysis data suggested that phosphorylated LATS1 level evoked by Nitidine chloride was reduced upon BEZ235 stimuli. Meanwhile, compared with phosphorylated LATS1 level, the expression of YAP and c-Myc were altered accordingly. Overall, the results indicated that BEZ235 interfered with the phosphorylated LATS1 level which evoked by Nitidine chloride, and resulted in the changes in c-Myc and YAP expression. Furthermore, in vivo, we found that the expression levels of YAP and c-Myc were increased in the BEZ235 treatment group than in the control group by immunohistochemical staining (Fig. 3D, E). These results confirmed that BEZ235 upregulated c-Myc and YAP expression in vivo and in vitro.

### YAP and c-Myc mediated resistance to the PI3K/mTOR dual inhibitor

**Fig. 4:**
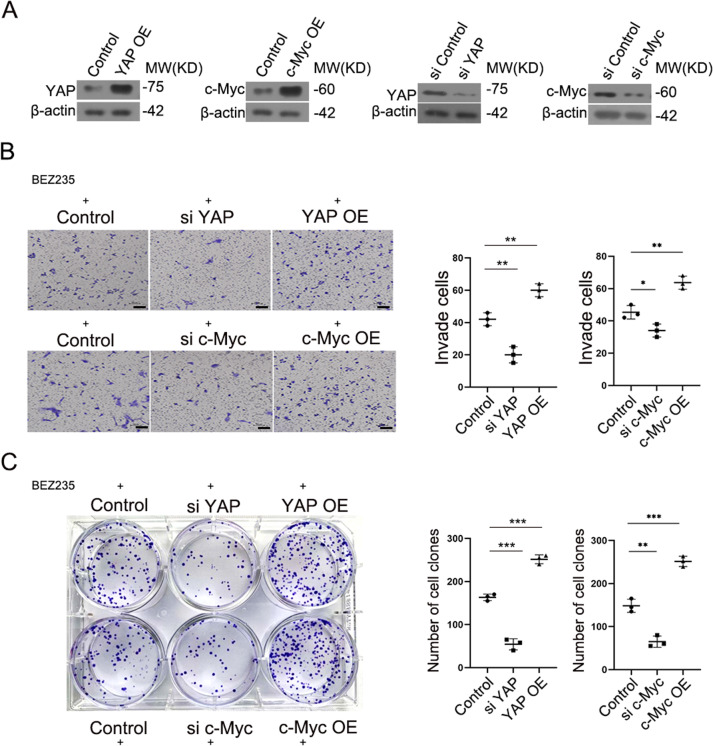
YAP and c-Myc mediated resistance to the PI3K/mTOR dual inhibitor. **A** The protein level of YAP and c-Myc were examined in RBE cells with YAP or c-Myc knockdown or overexpression by western blot. **B** Transwell assay and **C** colony formation assay showing the invasion potential of BEZ235-treatment cells that transfected with the distinct siRNA, plasmid. Magnification, ×100; scale bar, 100 μm. The data are shown as the mean ± SEM (*p < 0.05, **p < 0.01, ***p < 0.001).

RBE cells were transfected with YAP or c-Myc plasmids to overexpress the protein. Subsequently, RBE cells were transfected with siRNA control, siRNA YAP and siRNA c-Myc. We then detected the expression of YAP and c-Myc after transfection by western blotting (Fig. 4A). Transwell assays demonstrated that silencing YAP and c-Myc inhibited the invasion ability of BEZ235-treated RBE cells. Conversely, overexpression of YAP and c-Myc increased the invasion ability of BEZ235-treated RBE cells (Fig. 4B). As expected, BEZ235-treated HuCCT1 cells and QBC939 cells demonstrate the same conclusion by transwell assays (Supplemental Figs. 2B and 3B). This supports the conclusion that YAP and c-Myc increase BEZ235 resistance. Colony formation assays consistently confirmed that the overexpression of YAP and c-Myc increased BEZ235 resistance (Fig. 4C). We observed the same result on the colony formation assay across two additional ICC cell lines (Supplemental Figs. 2C and 3C).

### The combination of BET protein inhibition and PI3K/mTOR dual inhibition efficiently suppressed ICC progression in vitro

**Fig. 5:**
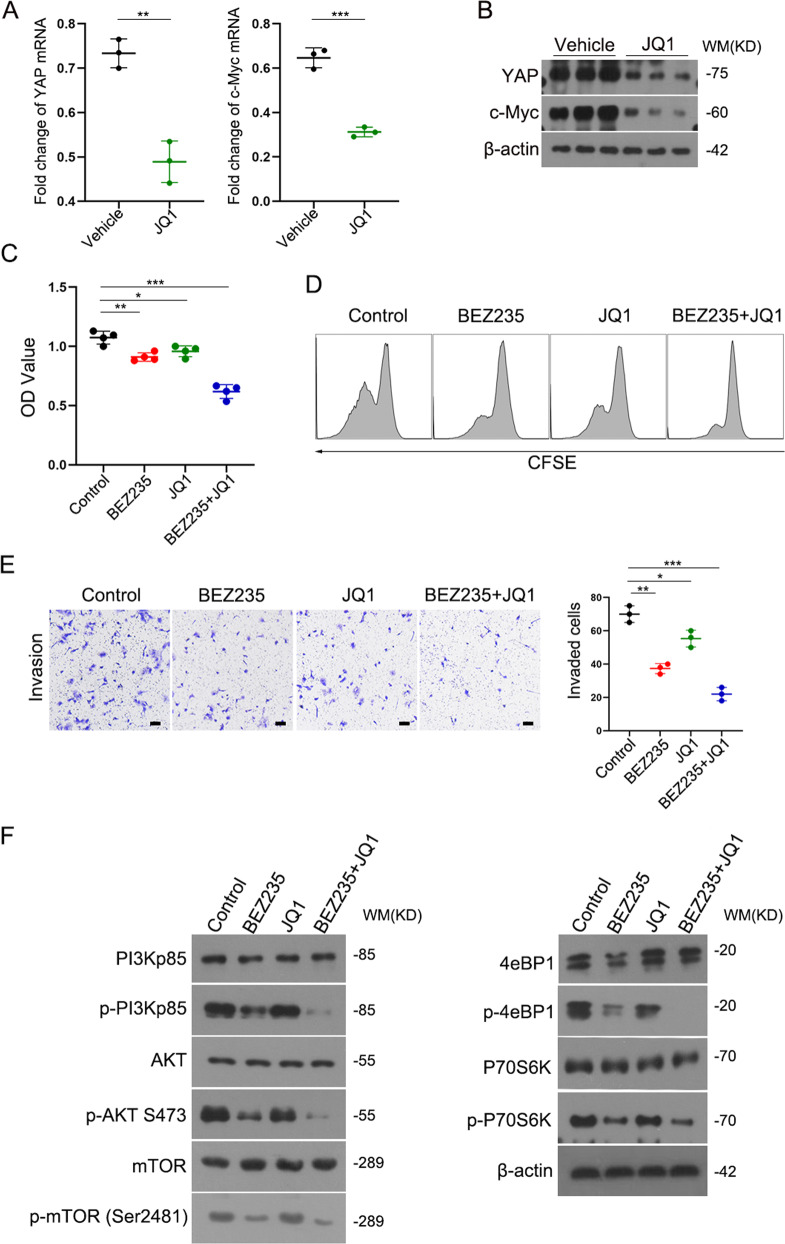
The combination of BET protein inhibition and PI3K/mTOR dual inhibition efficiently suppressed ICC progression in vitro. **A** Fold change in YAP and c-Myc mRNA expression level in RBE cells treated with DMSO or JQ1 (500 nM) for 24 hours. **B** western blot analysis of YAP and c-Myc protein levels in RBE cells treated with DMSO or JQ1 (500 nM) for 24 hours. **C** CCK-8 assay and **D** CFSE assay showing the cell proliferation ability in control, BEZ235, JQ1, and combination treatment groups. **E** Transwell assay showing the invasion potential of the four treatment groups. Magnification, ×100; scale bar, 100 μm. **F** RBE cells was incubated with DMSO, BEZ235 (100 nM), JQ1 (500 nM) and combination treatment for 24 hours, respectively. The cell lysates were gathered and the designated proteins (PI3Kp85, p-PI3Kp85, AKT, p-AKT (Ser473), mTOR, p-mTOR (Ser2481), 4eBP1, p-4eBP1, P70S6K and p-P70S6K) were detected by western blot analysis. The data are shown as the mean ± SEM (*p < 0.05, **p < 0.01, ***p < 0.001).

Given that JQ1-mediated inhibition of BRD4 decreased the levels of YAP and c-Myc [15, 18], we further detected the expression of YAP and c-Myc in JQ1-treated RBE cells. RBE cells were treated with DMSO or JQ1 (500 nM) for 24 hours. As expected, the mRNA and protein levels of YAP and c-Myc were significantly reduced in the JQ1 treatment group compared with the control group (Fig. 5A, B). Likewise, the protein level of YAP and c-Myc were also notably reduced under the treatment of JQ1 in HuCCT1 cells and QBC939 cells (Supplemental Figs. 2A and 3A). CCK-8 assays showed that the combination of JQ1 and BEZ235 significantly inhibited RBE cell proliferation compared to the control group or BEZ235 group (Fig. 5C). Compared with the result obtained from the CCK8 assay in the RBE cell line, the combination of JQ1 and BEZ235 also significantly inhibited HuCCT1 and QBC939 cell proliferation (Supplemental Figs. 5A and 6A). CFSE assays showed a similar result to CCK-8 assays, in which the combination of JQ1 and BEZ235 significantly inhibited RBE cell proliferation. Cell proliferation was further validated using EdU assay under the combination of JQ1 and BEZ235 treatment in two additional ICC cell lines. The EdU assay showed a lower percentage of EdU-positive (proliferating) cells under the combination of JQ1 and BEZ235 treatment compared to the control group or BEZ235 group (Supplemental Figs. 5B and 6B). In addition, Transwell assays were performed to detect cell invasion. The combination of JQ1 and BEZ235 exhibited significantly decreased effects in three ICC cell lines. (Fig. 5E, Supplemental Figs. 5C and 6C). We examined a protein concentration gradient (Supplemental Fig. 7A) and time point experiments (Supplemental Fig. 7B). RBE cells, HuCCT1 cells and QBC939 cells were separately treated with DMSO, BEZ235 (100 nM), JQ1 (500 nM), BEZ235 (100 nM) and JQ1 (500 nM) for 24 hours, and the protein levels were measured by western blot. The results showed that the combination of JQ1 and BEZ235 inhibited the expression of p-PI3K, p-AKT, p-mTOR, p-p70S6K and p-4eBP1 compared to the control group or BEZ235 group (Fig. 5F, Supplemental Figs. 5D and 6D). Therefore, a combination of JQ1 and BEZ235 more efficiently inhibited ICC progression in vitro.

### The combination of BET protein inhibition and PI3K/mTOR dual inhibition efficiently suppressed ICC progression in vivo

**Fig. 6:**
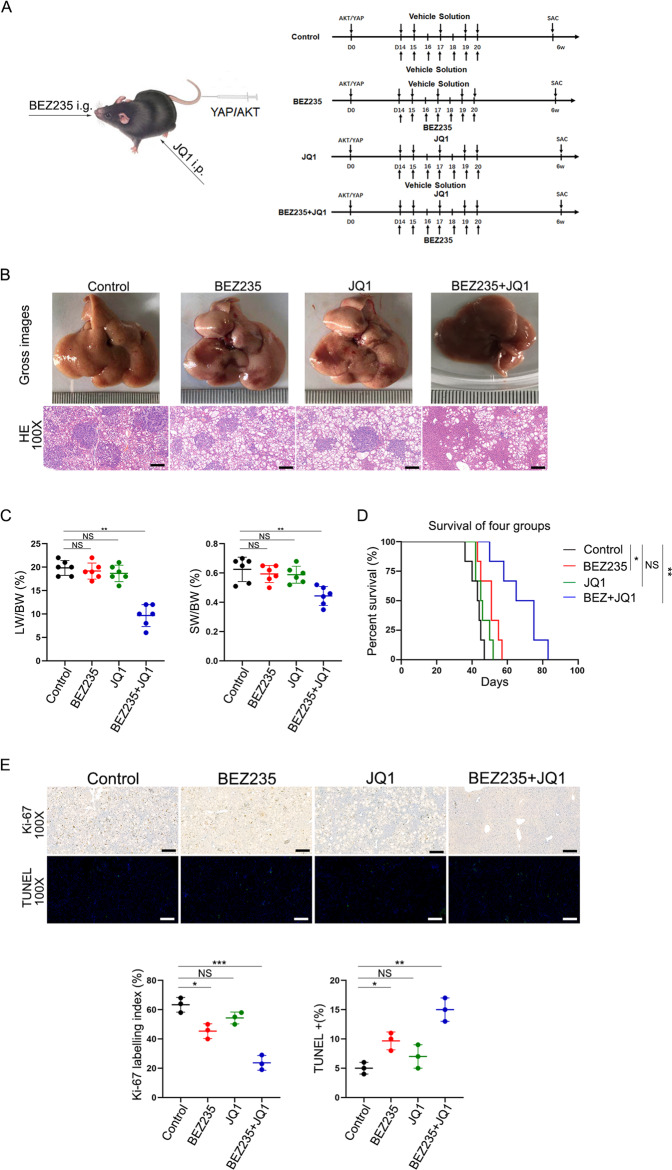
The combination of BET protein inhibition and PI3K/mTOR dual inhibition efficiently suppressed ICC progression in vivo. **A** Schematic of the experimental procedure for BEZ235, JQ1, or the combination treatment. The plasmids were transfected into all mice at day 0. JQ1 (50 mg/kg) (or vehicle solution) was i.p. injected at 14–20 days, and BEZ235 (30 mg/kg) (vehicle solution) was i.g. treated at 14–20 days, twice a day. All mice were sacrificed 6 weeks after oncogene transfection for phenotypic analysis. **B** Gross images, H&E staining of livers from four AKT/YapS127A mice groups. Magnification, ×100; scale bar, 100 μm. **C** Tumor burdens were calculated by LW/BW ratio, SW/BW ratios. The data are expressed as the means ± S.E.M (*n* = 6 per group, NS, *P* ≥ 0.05, **p < 0.01) for any other groups versus the control group. **D** Mouse Kaplan–Meier survival curve of the 4 treatment groups. Quantified data are presented as mean ± SE (NS, *P* ≥ 0.05, *P < 0.05, **p < 0.01). **E** immunohistochemical or immunofluorescence staining images of Ki-67 and TUNEL in liver sections to assess the tumor proliferation. Magnification, ×100; scale bar, 100 μm. The data are shown as the mean ± S.E.M (NS, *P* ≥ 0.05, *p < 0.05, **p < 0.01, ***p < 0.001).

The experimental strategy for drug administration is shown in Fig. 6A. AKT/YAP-transfected mice were treated with BEZ235, JQ1 or a combination of BEZ235 and JQ1 starting 2 weeks after oncogene transfection. The combination of BEZ235 and JQ1 significantly suppressed tumor progression, as evaluated by macroscopic view and H&E staining or by the LW/BW ratios and spleen weight/body weight (SW/BW) ratios (Fig. 6B, C). Moreover, compared to the survival time of the control group, the combination group showed a significant increase in survival time (Fig. 6D). Compared to the control group and either the BEZ235 or JQ1 group, the combined treatment significantly decreased the Ki67+ ratios in the tumor areas but increased TUNEL staining (Fig. 6E). Overall, these data demonstrate that combined treatment with JQ1 and BEZ235 effectively suppresses the progression of AKT/YAP S127 A ICC in mice.

### The effect of combination therapy on the tumor immune microenvironment

**Fig. 7:**
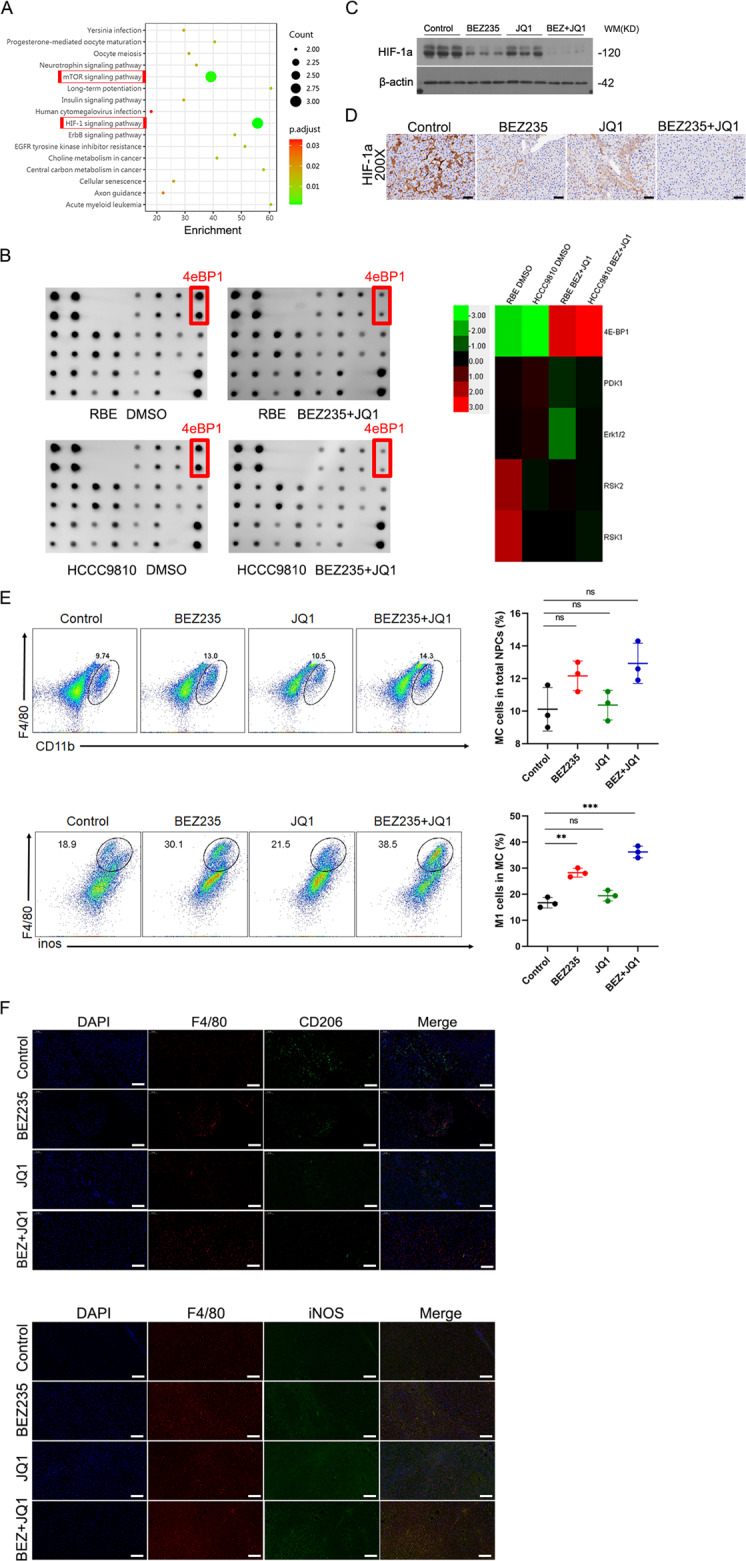
The effect of combination therapy on the tumor immune microenvironment. **A** KEGG pathway analysis showing the activated/phosphorylated protein in RBE cells treated with combination therapy or DMSO for 24 hours. **B** Left: Peptide phosphorylation microarray chips showing phospho-proteins differentially expressed in RBE and HCCC9810 cell lines following DMSO and combination therapy. Right: Heatmap showing phospho-proteins differentially altered in in RBE and HCCC9810 cell lines following DMSO and combination therapy (ANOVA, *P* < 0.05). Data are median centered (red: greater than the median, green: less). **C** Western blot analysis and **D** immunohistochemical staining of HIF-1α protein levels in ICC tissues derived from four treatment groups (control group, BEZ235 group, JQ1 group, combination group). Magnification, ×200; scale bar, 50 μm. **E** Representative flow cytometry dot plots and the percentages of MC cells in the total NPC population. **F** Representative flow cytometry dot plots and the percentages of M1 type macrophages in the total macrophages population. The data are shown as the mean ± S.E.M (NS, *P* ≥ 0.05, **p < 0.01, ***p < 0.001). **G**, **H** Immunofluorescence assay showing the expression of iNOS and CD206 in ICC tissues derived from four treatment groups (control group, BEZ235 group, JQ1 group, combination group). Magnification, ×200; scale bar, 50 μm.

In addition to the mTOR signaling pathway, HIF-1 signaling was the most significantly affected pathway by KEGG pathway analysis (Fig. 7A). Previous studies reported that the phosphorylation of 4EBP1 ultimately led to 4EBP1 binding to eIF4E and prevented protein synthesis [23, 24]. We further monitored the phosphorylation protein of the PI3K-AKT-mTOR signaling pathway in the two ICC cell lines between the combination-treated groups and DMSO-treated groups (Fig. 7B). mTOR plays a central role in the PI3K/AKT signaling pathway that regulates the translation of HIF-1α [25], whereas p-4E-BP1 is a direct target of mTOR [26]. We found that the phosphorylation of 4EBP1 was significantly reduced in both combination-treated groups. Therefore, we hypothesize that the combination of BEZ235 and JQ1 regulates the HIF-1 pathway by directly reducing 4EBP1 phosphorylation. To confirm this notion, we selected mouse liver tumor samples from 4 experimental groups (control group, BEZ235 group, JQ1 group, combination group) and extracted total protein for western blotting analysis (Fig. 7C). The protein expression of HIF-1α was also detected by immunohistochemical staining (Fig. 7D). The results supported the findings of the protein microarray, which showed that the group receiving a combination of JQ1 and BEZ235 had significantly inhibited expression of HIF-1α compared to the control group or single-drug group. Hypoxia-inducible factor-1a (HIF-1α) enhances liver cancer progression by inducing M2 polarization and suppressing M1 polarization in macrophages [27]. AKT/YAP S127A ICC mice treated with BEZ235, JQ1 or a combination of BEZ235 and JQ1, as shown in Fig. 6A, were sacrificed 2 days after the last dose of JQ1 or BEZ235 administration. Nonparenchymal cell (NPC) perfusates were collected using in situ liver perfusion. The ratio of the total number of macrophages to the number of NPCs was not significantly altered in the four experimental groups (Fig. 7E). Importantly, compared to the control group or single-drug group, the ratio of M1 macrophages to the total number of macrophages was significantly increased in the combination group (Fig. 7E). We further confirmed our findings with immunofluorescence staining of M1- and M2-type macrophages in liver tissue (Fig. 7F). Given the complexity of small-molecule inhibitors in the tumor immune microenvironment, combination therapy might be indicated to efficiently suppress the progression of ICC by inducing M1 polarization and suppressing M2 polarization.

## Discussion

Intrahepatic cholangiocellular carcinoma (ICC) is a fatal malignancy with a poor prognosis and only very limited therapeutic options. Consequently, there is an urgent need to identify new drug targets and develop effective therapeutic strategies for ICC. The PI3K/Akt/mTOR signaling pathway is frequently activated in many solid tumors and is therefore a major drug target for anticancer therapy [28–31]. The activation of the PI3K/Akt/mTOR pathway is involved in cell proliferation, cell migration and invasion and promotes cell apoptosis [32–34]. In the present study, we confirmed that mTOR signaling is the most significantly affected pathway in the human intrahepatic biliary epithelial cell line (HIBEpiC) and human cholangiocarcinoma cell line (RBE) by KEGG pathway enrichment analysis. Analyses of TCGA databases show the genetic alteration of PIK3CA and mTOR in liver cancer patients (Supplemental Fig. 8A). In addition, the expression of PIK3CA and mTOR also correlated with poor overall survival in liver cancer patients (Supplemental Fig. 8B). In recent years, there have been multiple efforts to focus on the development of mTOR inhibitors for cancer therapeutics.

As a novel PI3K/mTOR dual inhibitor, BEZ235 exerts antitumour activity by effectively and specifically blocking the dysfunctional activation of the PI3K/AKT/mTOR pathway. Our study confirmed that BEZ235 can suppress the proliferation, invasion and colony formation abilities of ICC cells in vitro. Given that the novel PI3K/mTOR dual inhibitor exhibited effective treatment of ICC cells in vitro, we next tested the therapeutic effect in a murine model. However, our in vivo experimental results indicate that BEZ235 cannot effectively inhibit ICC progression in vivo, which is not in accordance with the in vitro results.

Currently, molecular targeted therapy has emerged as a promising treatment strategy for cancer. On the other hand, resistance to molecular-targeted therapy also occurs in cancer cells, which represents a major clinical challenge. Furthermore, recent studies have demonstrated that YAP mediates crosstalk between the Hippo and PI3K-TOR pathways [35–37]. Additionally, the upregulation of c-Myc could participate in resistance to molecular-targeted therapy [38, 39]. Many studies have demonstrated that YAP is a stimulator of c-Myc transcription [40, 41]. Analyses of TCGA databases showed high genetic alterations of c-Myc and YAP in liver cancer patients (Supplemental Fig. 8A), and the expression of c-Myc and YAP correlated with poor overall survival in liver cancer patients (Supplemental Fig. 8B). Further TCGA database analysis also showed a positive correlation with YAP and mTOR (Supplemental Fig. 8C). This crosstalk provides a potential therapeutic target for rational combination therapy in liver cancer.

Interestingly, YAP is phosphorylated and inhibited by LATS 1/2 kinase [42]. Our data show that the PI3K/mTOR dual inhibitor increased c-Myc and YAP expression by suppressing the phosphorylation of LATS1 (Figs. 3 and 4). This would be a novel mechanism that mediates resistance to PI3K/mTOR dual inhibitors. It is imperative for us to explore a more effective therapeutic strategy for ICC.

BET inhibition by JQ1 downregulates MYC transcription, which is a proto-oncogene overexpressed in cancer cells [15]. The YAP/transcriptional coactivator with PDZ-binding motif (TAZ) and Notch signaling pathways were reported to be suppressed by JQ1 in our previous study [18]. However, our work also did not exhibit effective treatment of ICC alone in vivo. In our study, we found that the combination of BEZ235 and JQ1 significantly suppressed tumor progression and increased survival times (Fig. 6). Indeed, the efficacy results of combination therapy exhibited effective treatment on ICC in vitro and in vivo.

The progression of solid tumors is in part influenced by the local inflammatory microenvironment [27]. Hypoxia is a feature of most tumors that plays a mediating role in tumor progression [43] and leads to distinct properties of TAMs [44]. However, hypoxia-inducible factor 1a (HIF-1α) plays a critical role in the function of tumor-associated macrophages (TAMs). M2-polarized TAMs drive tumor progression and invasion, while M1-polarized TAMs act as tumor suppressors [45]. mTOR plays an important role in regulating the translation of HIF-1α [25]. Therefore, we hypothesize that the combination of a PI3K/mTOR dual inhibitor and BET inhibition induces M1 polarization and suppresses M2 polarization in macrophages by regulating the expression of HIF-1α. As expected, our data indeed supported this hypothesis (Fig. 7).

Due to the unique immunotolerant microenvironment of the liver and the high expression of YAP, ICC is a unique cancer that is difficult to treat. We believe that this issue can be remedied by new drug combinations that improve antitumour activity and modulate the functions of tumor infiltrating immune cells in the liver. It has been reported that the combination of BEZ235 and JQ1 results in robust cell death in vitro and xenograft regression in vivo [46]. However, neither of these small-molecule inhibitors alone exhibited effective treatment in a xenograft tumor nude mouse model. We found that combined treatment with BEZ235 and JQ1 effectively suppresses the progression of primary intrahepatic cholangiocarcinoma in mouse models.

Current treatment options and emerging therapies, cannot effectively inhibit ICC progression. Our findings reveal a novel mechanism by which a dual PI3K/mTOR inhibitor suppresses the phosphorylation of LATS1 and therefore increases c-Myc and YAP expression, which mediates resistance to dual PI3K/mTOR inhibitors. The combination therapy improved antitumour activity and simultaneously modulated the functions of tumor-associated macrophages in the liver. Additionally, the combined treatment was identified as an effective therapeutic strategy for ICC. Nevertheless, the results of this new combination therapy were provided only by theoretical considerations. Further studies are necessary to determine the therapeutic effect of PI3K/mTOR dual inhibitors and BET inhibition on ICC in a clinical setting.

## Methods and materials

### Establishment of the murine ICC model and treatment

To generate the ICC model, hydrodynamic tail vein injection (HTVi) was performed as described [19]. Plasmids (pT3-EF1a-myrAKT-HA; pT3-EF1a-FLAG-Yap S27A) were purchased from *Addgene*. The plasmid (pCMVSB11) was a gift from Dr. Liang Wen at Zhejiang University. pT3-EF1a-myrAKT-HA (10ug), pT3-EF1a-FLAG-YAP S127A(10ug) and pCMVSB11(5ug) were diluted in 2.5 ml saline (0.9% NaCl) for each mouse, and then delivered to 8 weeks-old mice by hydrodynamic tail vein injection within 5 to 7 s. JQ1 (S7110, Selleckchem) was injected intraperitoneally (i.p.) at 50 mg/kg for five doses at the indicated dates. BEZ235 (S1009, Selleckchem) was orally administered via gavage at 30 mg/kg at the indicated dates. Groups allocation for the experiments was randomized and not blinded. Sample analyses were not blinded. Animal experiments were approved by the Animal Care Committee of Zhejiang University and were performed in compliance with the Animal Management Rules of the Chinese Ministry of Health (Document No. 55, 2001).

### RNA-sequencing and transcriptome analysis

RBE cell lines were harvested for total RNA extraction using TRIzol reagent (Invitrogen) after being treated with drugs for 24 h. The samples were further purified using an mRNA purification kit (Invitrogen) and then sent to Shanghai Majorbio Bio-Pharm Technology Co., Ltd for transcriptome sequencing by Illumina HiSeqTM 2500 sequencer. Based on the following criteria: OD 260/A280 ≈ 2.1, OD 260/230 ≈ 2.0, quantity> 15 μg. The data were analyzed on the free online platform of Majorbio I-Sanger Cloud Platform (www.i-sanger.com).

### Statistical analysis

SPSS v23 (SPSS Inc., Chicago, IL) was used for experimental data analysis and data passed normality and equal variance tests. All experiments were independently repeated at least three times. The sample size was calculated by using PASS 11 (NCSS Inc). Statistical comparisons between 2 groups involved Student’s *t* test and otherwise one-way ANOVA and Bonferroni post-tests. All data are expressed as the mean ± standard error of the mean (SEM). All statistical tests were two-tailed, and *P* < 0.05 was considered statistically significant.

## Supplementary information

Supplemental document

Supplemental Fig. 1

Supplemental Fig. 2

Supplemental Fig. 3

Supplemental Fig. 4

Supplemental Fig. 5

Supplemental Fig. 6

Supplemental Fig.7

Supplemental Fig. 8

## Data Availability

All data generated in the study are included in this article.
