# Supplementary material for: BET protein inhibition evidently enhances sensitivity to PI3K/mTOR dual inhibition in intrahepatic cholangiocarcinoma
Source: Cell Death Dis. 2021 Oct 29;12(11):1020. doi: 10.1038/s41419-021-04305-3 (PMC8556340; doi:10.1038/s41419-021-04305-3)
Supplement: Supplementary file 1 — Supplemental document [file 41419_2021_4305_MOESM1_ESM.docx]

**Methods and Material**

**Animal Studies**

Male C57BL6/J mice (six weeks old) were purchased from Beijing Vital River Laboratory Animal Technology Co., Ltd. (Beijing, China). The animal experiment was approved by the Institutional Animal Care and Use Committee of Zhejiang University. All mice were housed under specific pathogen-free conditions on a 12-h light/12-h dark cycle with food and water freely available. All procedures conformed to the guidelines from the NIH Guide for the Care and Use of Laboratory Animals.

**Phospho-antibody array analysis**

To analyse the phosphorylation state of the Akt pathway we used the Human/Mouse AKT Pathway Phosphorylation Array C1 from RayBiotech (AAH-AKT-1-8, Norcross, GA). Cells were seeded in 100-mm dishes and incubated at 37 °C for 24 hours. Cells were washed twice with cold phosphate-buffered saline, followed by solubilization at 2 x 10^7^ cells/mL in 1 x lysis buffer provided by Raybiotech (Norcross, GA). Cell lysates were incubated on ice for 30 minutes and then centrifuged at top speed, and protein was quantified using the Pierce BCA Protein assay. Relative levels in cell lysates (500μg per sample) were analyzed and incubated with the phospho-kinase array membrane according to manufacturer's protocol. The experimental operation was conducted by Raybiotech (Guangzhou, China).

**Reagents**

The antibodies used were specific for total PI3K (4257, Cell Signaling Technology), phospho-PI3K (4228, Cell Signaling Technology), total Akt (4691, Cell Signaling Technology), phospho-Akt (Ser473) (4060, Cell Signaling Technology), total mTOR (2983, Cell Signaling Technology), phospho-mTOR (Ser2448) (2971, Cell Signaling Technology), total p70S6K (2708, Cell Signaling Technology), phospho-p70S6K (Thr389) (9234, Cell Signaling Technology), total 4E-BP1 (9644, Cell Signaling Technology), phospho-4E-BP1 (Thr37/46) (2855, Cell Signaling Technology), total LATS1 (3477, Cell Signaling Technology), phospho- LATS1(T1079) (8654S, Cell Signaling Technology), YAP (14074, Cell Signaling Technology), c-Myc (5605, Cell Signaling Technology), RHEB (ab25873, Abcam), S6K1(ab32359), Hif-1α (ab1, Abcam). Adenoviral constructs to overexpress Flag-tagged YAP, Flag-tagged c-Myc and GFP control were from ViGene BioSciences (Jinan, China). Control small interfering RNA, YAP siRNA and c-Myc siRNA were also from ViGene BioSciences (Jinan, China). BEZ235 (S1009) and JQ1 (S7110) were from Selleckchem. Nitidine chloride was obtained from Tauto Biotech Company (Shanghai, China).

**Cell culture**

Human cholangiocarcinoma cell line (RBE) was provided by Stem Cell Bank of the Chinese Academy of Sciences. Human intrahepatic biliary epithelial cell line (HIBEpiC) was purchased from ATCC. RBE cells and HIBEpiC cells were cultured in Dulbecco's modified Eagle medium (DMEM) containing 10% fetal bovine serum (FBS; BI, USA), penicillin, streptomycin (15070063, Thermo Fisher) at 37 °C in 5% CO2. Human cholangiocarcinoma cell line (HCCC9810) was kindly provided by Procell Life Science & Technology CO.,Ltd. HCCC9810 cells were maintained at 37°C with 5% CO2 in Roswell Park Memorial Institute (RPMI-1640,Gibco) supplemented with 10% fetal bovine serum (FBS; BI, USA), penicillin, streptomycin (15070063, Thermo Fisher). Human cholangiocarcinoma cell line (HuCCT1 cells and QBC939 cells) were kindly provided by Procell Life Science & Technology CO.,Ltd. HuCCT1 cell and QBC939 cells were maintained at 37°C with 5% CO2 in Roswell Park Memorial Institute (RPMI-1640,Gibco) supplemented with 10% fetal bovine serum (FBS; BI, USA), penicillin, streptomycin (15070063, Thermo Fisher).

**Histopathology**

To analyze histopathological alterations in ICC, liver tissues were arrested and fixed in 10% formalin (SF98-4; Fisher) at 4 °C until use. The fixed tissues were dehydrated and processed for paraffin embedding, and 5-μm sections were stained with hematoxylin and eosin (H&E).

**Immunohistochemical staining (IHC) and Immunofluorescence**

Briefly, deparaffinized and rehydrated sections were heated in citrate buffer at 121°C for 30 min, incubated with 0.3% hydrogen peroxide in methanol for 20 min and blocked with 10% normal bovine serum. The slides were incubated with rabbit polyclonal antibodies at 4°C overnight, followed by incubation with Alexa Fluor-conjugated secondary antibody (Life Technologies) diluted in block buffer for 1h at room temperature. Slides were examined by using a laser scanning confocal microscope (Zeiss LSM 800).

**Cell proliferation assays**

Cell proliferation was assessed by EdU assay, CFSE assay, Cell Counting Kit-8 assay and Colony formation assay. The EdU assay kit, CFSE assay kit and CCK-8 kit were all purchased from Beyotime Biotechnology Company.

For CFSE assay, in brief, 4 × 10^7^ cells were seeded in well plates and labeled with 0.5 µM CFSE at 37°C for 10 min. As the cell cultures were expanded, levels of CFSE staining declined with each cell division. CFSE proliferation was assessed by flow cytometry.

EdU detection was performed using the EdU-Click Chemistry 488 kit according to manufacturer’s instructions. Briefly, 1 × 10^5^ cells were pulsed with 10 μM EdU alongside simultaneous treatment with DMSO. Cells were fixed, washed with PBS, blocked. Then cells were incubated at RT for 30 minutes in the click chemistry cocktail. The nuclei were stained with DAPI. Fluorescence microscopy was used to observe the EdU positive cells and Image J software was used for further analysis.

Cells were seeded into 96-well plates with 2000 cells/well together with 10 μL CCK-8 solution and cultured for 2 h. The optical density (OD) were subsequently measured at 450 nm and 630 nm.

The colony formation assay was performed using a 6-well plate with 1 × 10^5^ cells/well. The cells were seeded in the culture plates for about 2 weeks until the cloned cells were seen macroscopically. After 4 weeks, colonies were stained with 0.05% crystal violet. After staining, cell colony number was measured using ImageJ software.

**Cell migration and invasion assays**

The transwell assay was used to assess the migration and invasion ability. Martrigel was coated on polycarbonate membrane of the upper chambers. RBE cell and HCCC9810 cell (5×10^4^ cells/well) were inoculated with 200 μl cell suspension (FBS-free medium) in the upper chambers, while the lower chambers added in 600 μl of 10% fetal bovine serum medium. Then cultured in 37°C for 16-24 hours. After fixed with 4% para­formaldehyde for 20 min and stained with 0.1% crystal violet for 5 min, the invasive cells were counted in three random fields under a microscope.

**TUNEL assay**

Mouse liver tissues that received four treatments were used for TUNEL assay. 4mm frozen sections were washed in PBS for 5 min and then incubated with proteinase K for 3 min at room temperature. After incubation with equilibration buffer for 30 min, the TUNEL reaction mixture was added to rinsed slides. Slides were washed in PBS and labeled with the Live/Dead Fluorescein TUNEL kit (Promega) according to the manufacturer’s protocol.

**Western Blot Analysis**

Protein extracts were prepared by lysing tissues or cells in RIPA lysis buffer (89900; Thermo Scientific) supplemented with protease inhibitor (B14001; Bimake) and complete mini proteasome inhibitors (05892791001; Sigma-Aldrich), fractionated by SDS-PAGE, then transferred onto PVDF membranes. After blocking in 5% skim milk, blots were probed with specific primary antibodies at 4℃ overnight. After 3 cycles of cleaning with TBST, membranes were incubated with appropriate horseradish peroxidase-conjugated secondary antibodies and observed by enhanced chemiluminescence (Pierce). ImageJ was used to determine the quantification of western blot band intensity.

**Cell isolation and flow cytometric analysis**

Mice liver in situ perfusion was performed using a solution containing 0.08% collagenase II and IV (Gibco) for 30 min. Isolated cells were centrifuged at 70 g for 5 min to collect primary hepatocytes. The supernatant was centrifuged at 300 g for 5 min to collect the nonparenchymal cells (NPCs). They were blocked, stained, and analyzed using FACSCalibur (BD) and FlowJo software (Tree Star). The following mAbs were used: F4/80 (123110) was purchased from all from Biolegend (San Diego, USA). CD16/32 (553142), CD11b (557396) were from BD Biosciences (San Jose, USA). F4/80 (2054484), iNOS (2088487), CD206(2005201) were from Invitrogen (Carlsbad, CA).

**Supplemental Figure Legends:**

1. Supplemental Fig. 1 (A) colony formation assay showing the cell proliferation ability in RBE cells treated with DMSO or BEZ235 (100 nM) for 24 hours.

2. Supplemental Fig. 2 (A) western blot analysis of YAP and c-Myc protein levels in HuCCT1 cells treated with BEZ235 (100 nM) or JQ1 (500 nM) for 24 hours. (B) Transwell assay and (C) colony formation assay showing the invasion potential of BEZ235-treatment cells that transfected with the distinct siRNA, plasmid. Magnification, ×100; scale bar, 100 μm. The data are shown as the mean ± SEM (*p < 0.05, **p < 0.01, ***p < 0.001).

3. Supplemental Fig. 3 (A) western blot analysis of YAP and c-Myc protein levels in QBC939 cells treated with BEZ235 (100 nM) or JQ1 (500 nM) for 24 hours. (B) Transwell assay and (C) colony formation assay showing the invasion potential of BEZ235-treatment cells that transfected with the distinct siRNA, plasmid. Magnification, ×100; scale bar, 100 μm. The data are shown as the mean ± SEM (*p < 0.05, **p < 0.01, ***p < 0.001).

4. Supplemental Fig. 4 (A) The expression of p-LATS1 was detected in Nitidine chloride-treated RBE cells and DMSO-treated RBE cells by western blotting for screening. (B) RBE cells was incubated with DMSO, BEZ235 (100 nM), Nitidine chloride (4 uM) and combination treatment for 24 hours, respectively. The cell lysates were gathered and the designated proteins (YAP, c-Myc, LATS1 and p- LATS1) were detected by western blot analysis.

5. Supplemental Fig. 5 (A) CCK-8 assay and (D) EdU assay showing the cell proliferation ability in control, BEZ235, JQ1, and combination treatment groups. (B) Transwell assay showing the invasion potential of the four treatment groups. Magnification, ×100; scale bar, 100 μm. (C) HuCCT1 cells was incubated with DMSO, BEZ235 (100 nM), JQ1 (500 nM) and combination treatment for 24 hours, respectively. The cell lysates were gathered and the designated proteins (PI3Kp85, p-PI3Kp85, AKT, p-AKT (Ser473), mTOR, p-mTOR (Ser2481), 4eBP1, p-4eBP1, P70S6K and p-P70S6K) were detected by western blot analysis. The data are shown as the mean ± SEM (*p < 0.05, **p < 0.01, ***p < 0.001).

6. Supplemental Fig. 6 (A) Cell Counting Kit-8 assay and (B) EdU assay showing the cell proliferation ability in control, BEZ235, JQ1, and combination treatment groups. (B) Transwell assay showing the invasion potential of the four treatment groups. Magnification, ×100; scale bar, 100 μm. (C) HuCCT1 cells was incubated with DMSO, BEZ235 (100 nM), JQ1 (500 nM) and combination treatment for 24 hours, respectively. The cell lysates were gathered and the designated proteins (PI3Kp85, p-PI3Kp85, AKT, p-AKT (Ser473), mTOR, p-mTOR (Ser2481), 4eBP1, p-4eBP1, P70S6K and p-P70S6K) were detected by western blot analysis. The data are shown as the mean ± SEM (*p < 0.05, **p < 0.01, ***p < 0.001).

7. Supplemental Fig. 7 (A) RBE cell was incubated with BEZ235 (100 nM) for different time points. (B) RBE cell was incubated under various concentrations of BEZ235 for 24 h. The expression of p-PI3Kp85 and p-mTOR were detected by western blotting for screening respectively.

8. Supplemental Fig. 8 (A) Gene expression data from the TCGA data portal. (B) The overall survival analysis of TCGA database. (C) The correlations between mTOR and YAP mRNA expression in human liver cancer.
