# Supplementary figures and images for: BET protein inhibition evidently enhances sensitivity to PI3K/mTOR dual inhibition in intrahepatic cholangiocarcinoma
Source: Cell Death Dis. 2021 Oct 29;12(11):1020. doi: 10.1038/s41419-021-04305-3 (PMC8556340; doi:10.1038/s41419-021-04305-3)

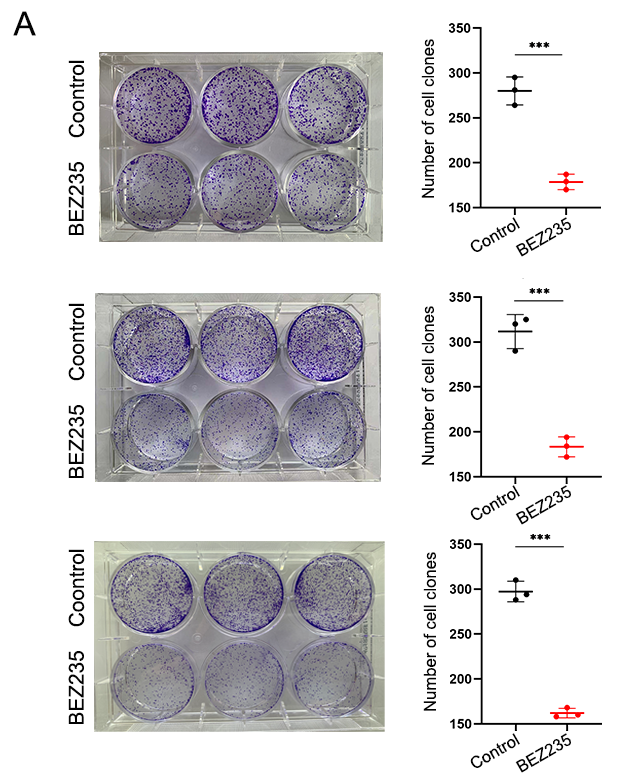

Supplement: Supplementary file 2 — Supplemental Fig. 1 [file 41419_2021_4305_MOESM2_ESM.tif]

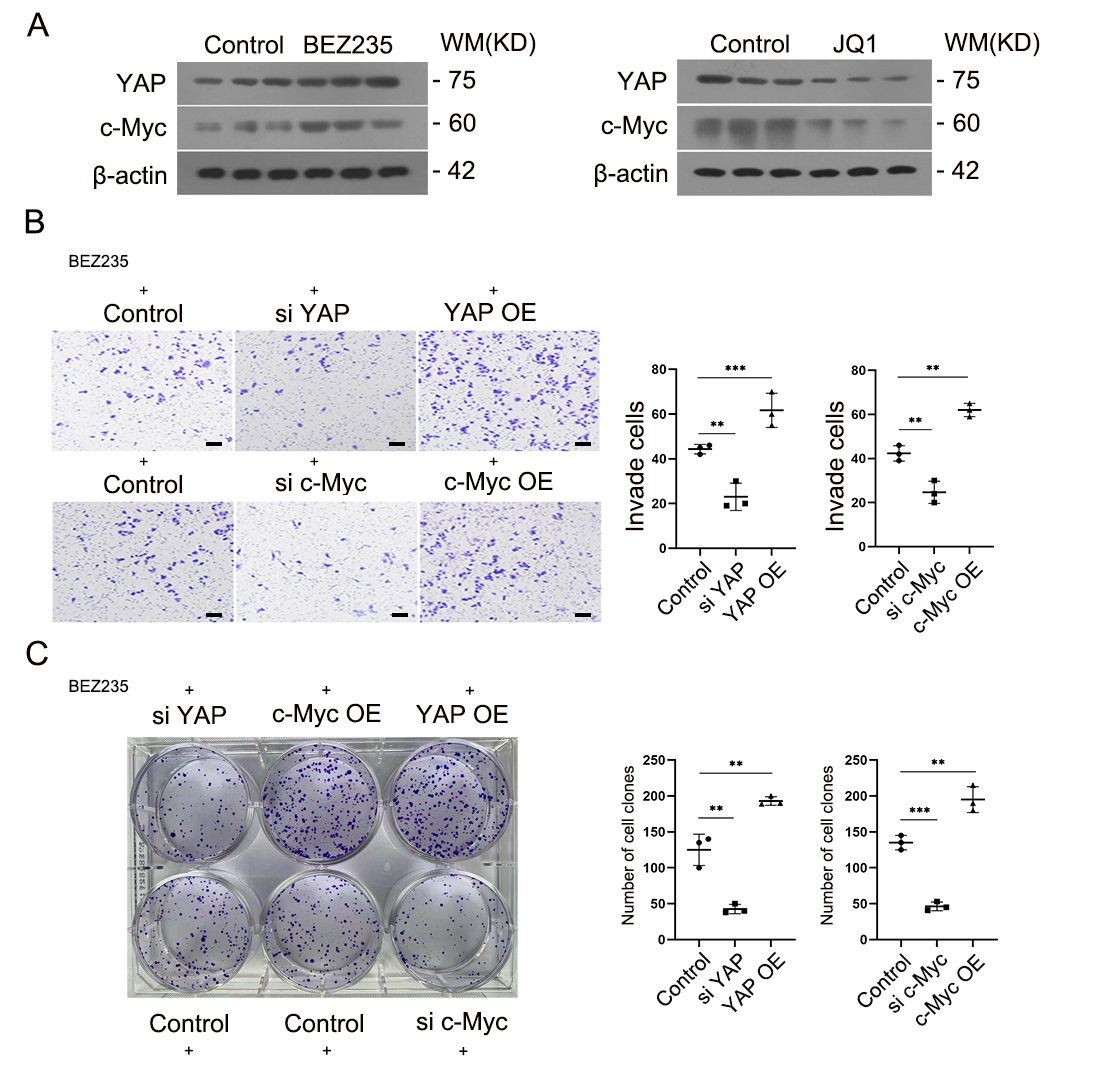

Supplement: Supplementary file 3 — Supplemental Fig. 2 [file 41419_2021_4305_MOESM3_ESM.tif]

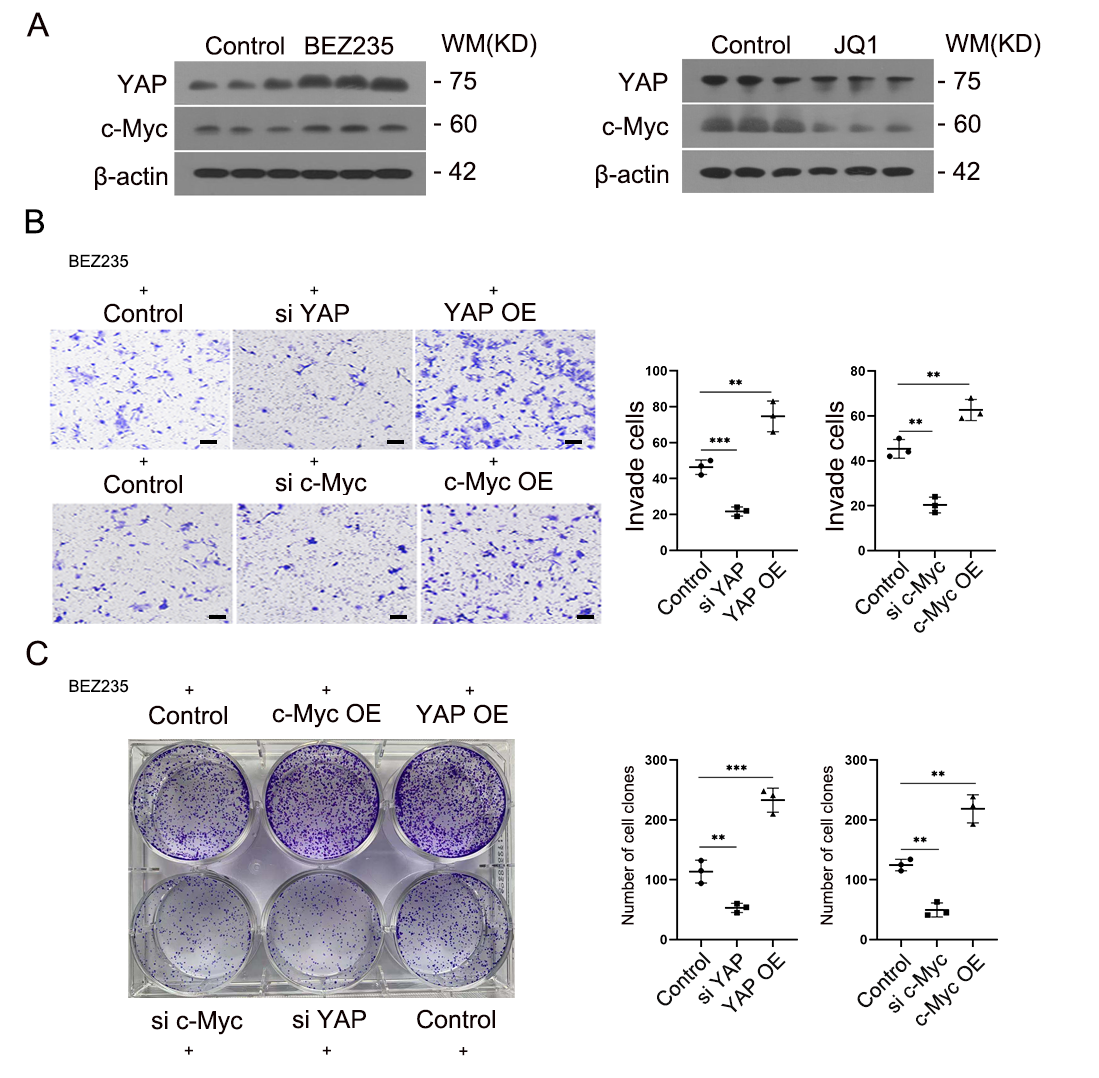

Supplement: Supplementary file 4 — Supplemental Fig. 3 [file 41419_2021_4305_MOESM4_ESM.tif]

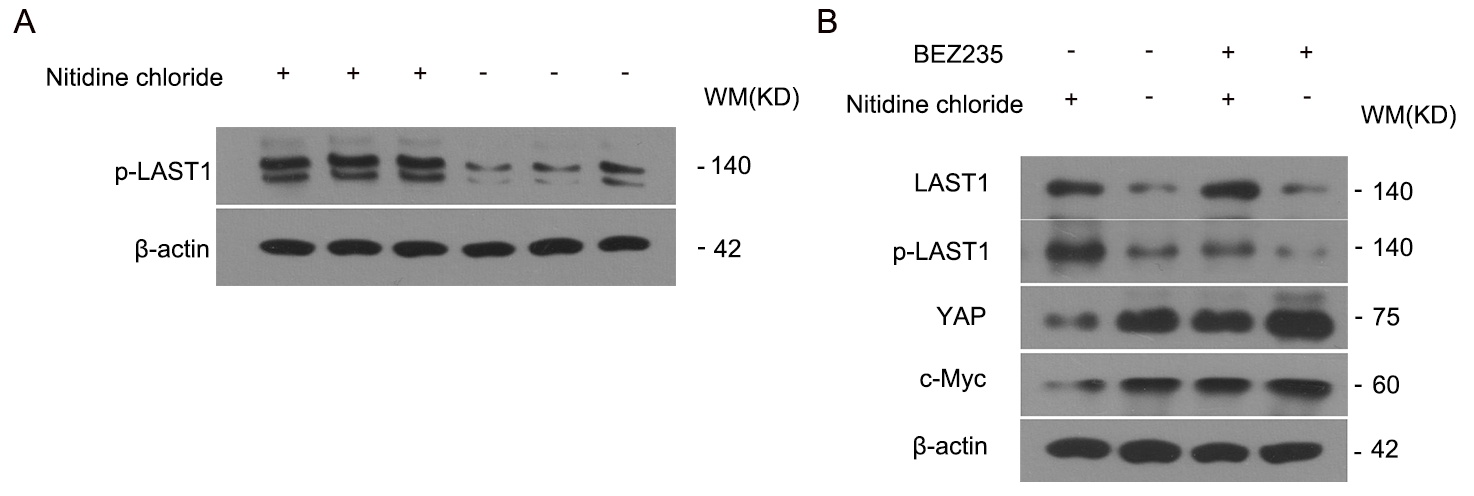

Supplement: Supplementary file 5 — Supplemental Fig. 4 [file 41419_2021_4305_MOESM5_ESM.tif]

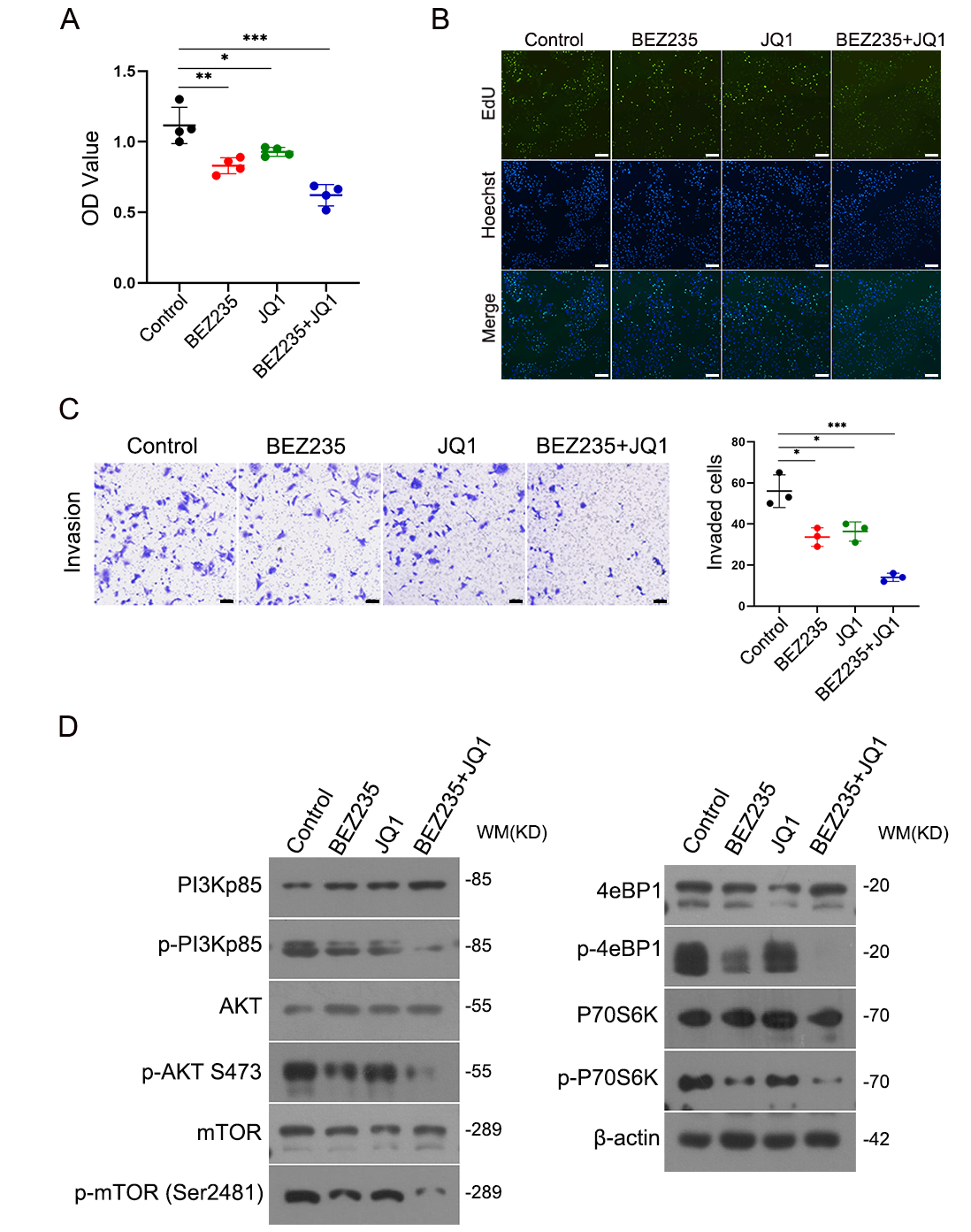

Supplement: Supplementary file 6 — Supplemental Fig. 5 [file 41419_2021_4305_MOESM6_ESM.tif]

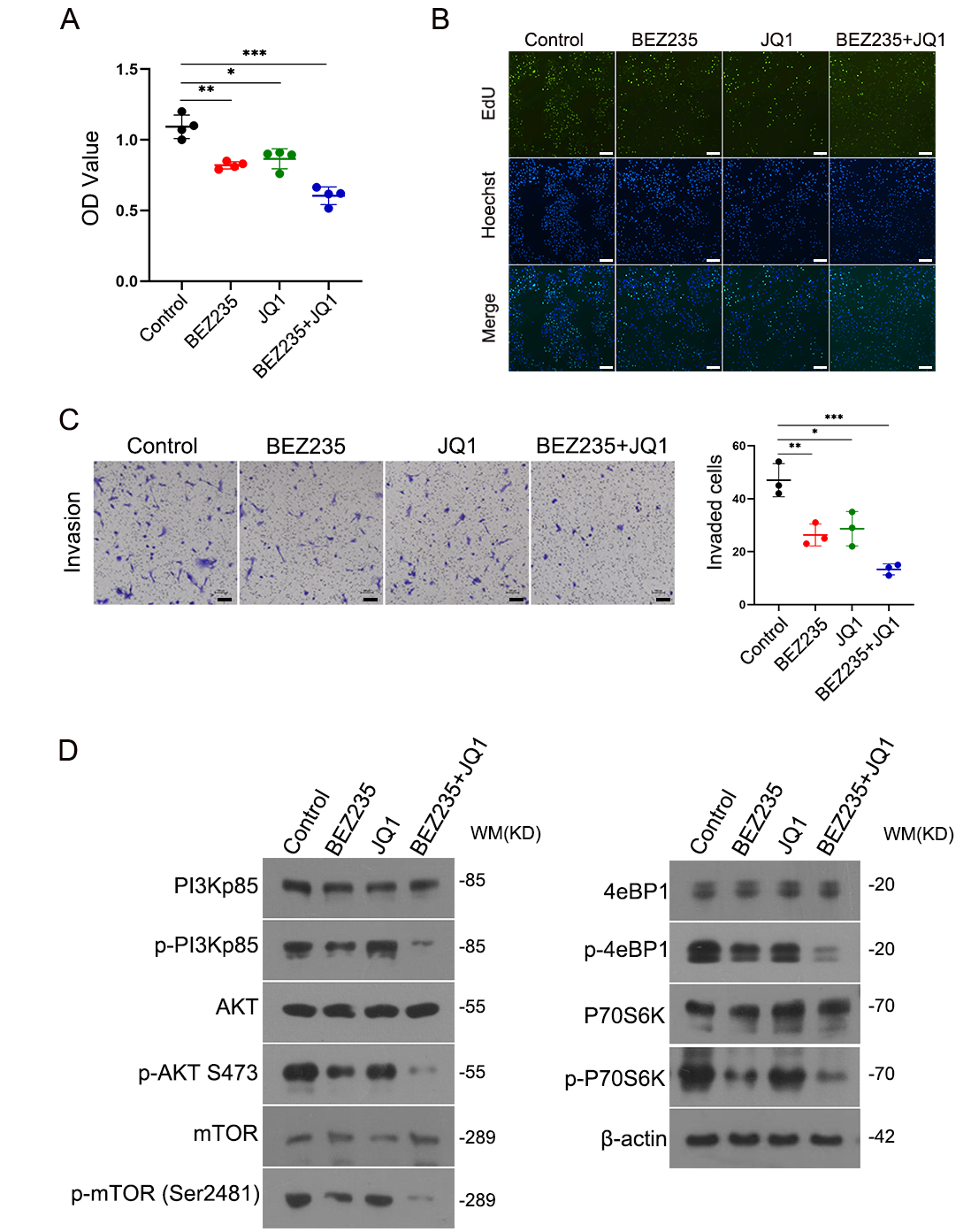

Supplement: Supplementary file 7 — Supplemental Fig. 6 [file 41419_2021_4305_MOESM7_ESM.tif]

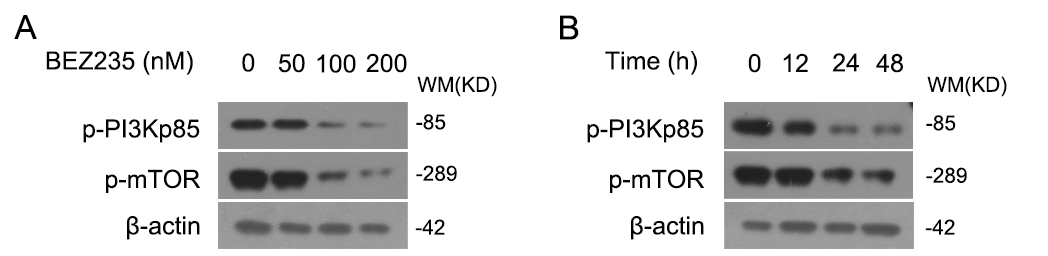

Supplement: Supplementary file 8 — Supplemental Fig.7 [file 41419_2021_4305_MOESM8_ESM.tif]

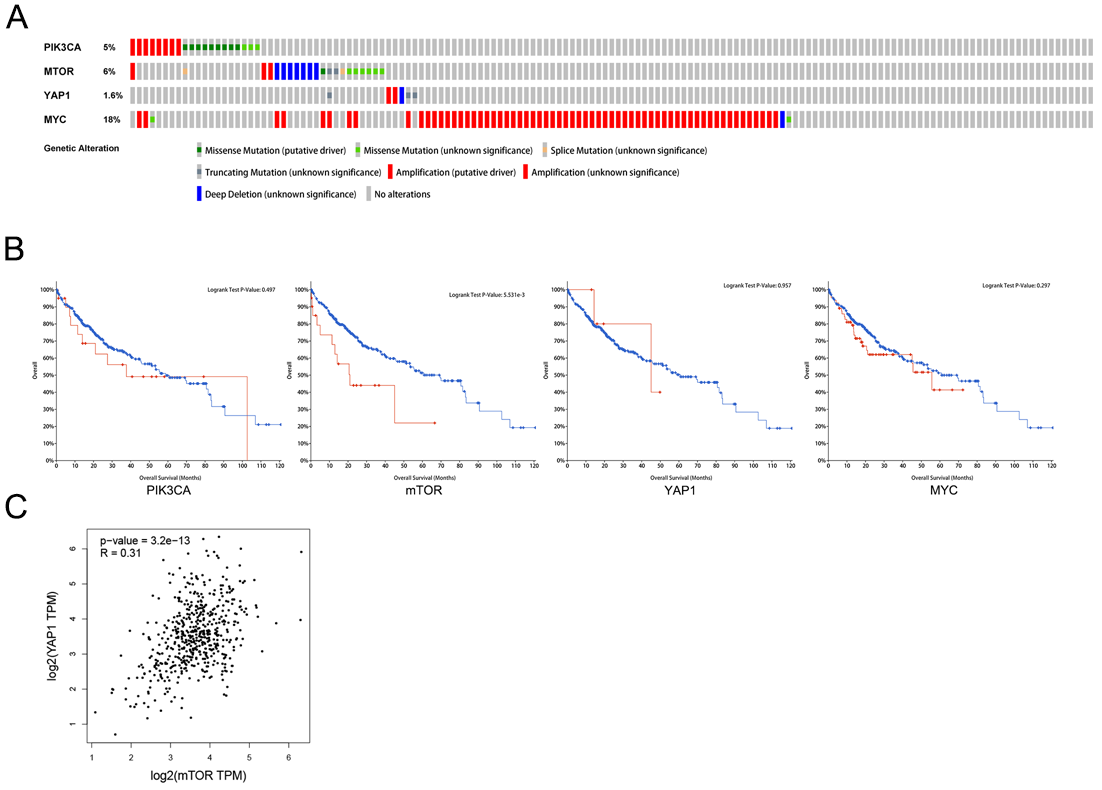

Supplement: Supplementary file 9 — Supplemental Fig. 8 [file 41419_2021_4305_MOESM9_ESM.tif]
